# Ageing-associated changes in DNA methylation in X and Y chromosomes

**DOI:** 10.1186/s13072-021-00407-6

**Published:** 2021-07-02

**Authors:** Laura Kananen, Saara Marttila

**Affiliations:** 1 Faculty of Social Sciences (Health Sciences), Tampere University, Tampere, Finland; 2 Faculty of Medicine and Health Technology, Tampere University, Tampere, Finland; 3 Department of Medical Epidemiology and Biostatistics, Karolinska Institutet, Stockholm, Sweden; 4 Gerontology Research Center, Tampere University, Tampere, Finland; 5 Department of Clinical Chemistry, Faculty of Medicine and Health Technology, Tampere University, Tampere, Finland

**Keywords:** Ageing, DNA methylation, Sex chromosomes, X chromosome, Y chromosome

## Abstract

**Background:**

Ageing displays clear sexual dimorphism, evident in both morbidity and mortality. Ageing is also associated with changes in DNA methylation, but very little focus has been on the sex chromosomes, potential biological contributors to the observed sexual dimorphism. Here, we sought to identify DNA methylation changes associated with ageing in the Y and X chromosomes, by utilizing datasets available in data repositories, comprising in total of 1240 males and 1191 females, aged 14–92 years.

**Results:**

In total, we identified 46 age-associated CpG sites in the male Y, 1327 age-associated CpG sites in the male X, and 325 age-associated CpG sites in the female X. The X chromosomal age-associated CpGs showed significant overlap between females and males, with 122 CpGs identified as age-associated in both sexes. Age-associated X chromosomal CpGs in both sexes were enriched in CpG islands and depleted from gene bodies and showed no strong trend towards hypermethylation nor hypomethylation. In contrast, the Y chromosomal age-associated CpGs were enriched in gene bodies, and showed a clear trend towards hypermethylation with age.

**Conclusions:**

Significant overlap in X chromosomal age-associated CpGs identified in males and females and their shared features suggest that despite the uneven chromosomal dosage, differences in ageing-associated DNA methylation changes in the X chromosome are unlikely to be a major contributor of sex dimorphism in ageing. While age-associated CpGs showed good replication across datasets in the present study, only a limited set of previously reported age-associated CpGs were replicated. One contributor to the limited overlap are differences in the age range of individuals included in each data set. Further study is needed to identify biologically significant age-associated CpGs in the sex chromosomes.

**Supplementary Information:**

The online version contains supplementary material available at 10.1186/s13072-021-00407-6.

## Background

Ageing displays clear sexual dimorphism. Females have a longevity advantage, which can be observed across nationalities and in historical data, where and when reliable demographic information is available [1, 2]. Despite the longer lifespan, females have poorer health in later life, the so-called mortality–morbidity paradox [3, 4]. Epidemiology, pathophysiology, and symptoms of ageing-associated diseases, such as cardiometabolic diseases, cancer, and neurodegenerative diseases, can also differ between males and females [3]. The mechanisms underlying this sexual dimorphism remain poorly understood, but contributing factors include both biological as well as behavioral and societal [1, 3, 5].

Sex chromosomes are one of the biological factors contributing to sexual dimorphism in humans and other mammals [6]. The Y chromosome is small (57 million base pairs, bp) and relatively gene poor, with 566 genes, majority of which are pseudogenes. For comparison, chromosomes 22 and 19 (51 and 59 million bp, respectively) contain 1376 and 2914 genes, respectively [7, 8]. The X chromosome is 156 million bp long and contains 2408 genes [7, 8]. Dosage compensation between XX females and XY males is achieved via X chromosome inactivation (XCI) in females, where one of the X chromosomes is packaged as heterochromatin and is nearly fully methylated. XCI occurs at random at an early developmental stage, leading to mosaic expression from both maternal and paternal X. In addition, a proportion of X chromosomal genes escape XCI [6].

Ageing-associated changes in DNA methylation have been widely studied (for example [9–12], see also references in “Discussion”), but in the great majority of studies, focus has been on the autosomes and sex chromosomes have been excluded. Here, we sought to identify DNA methylation changes associated with ageing in the Y and X chromosomes, by utilizing five datasets, all of whole blood, available in data repositories, comprising in total of 1240 males and 1191 females, aged 14–92 years.

## Results

### Y chromosome

**Fig. 1:**
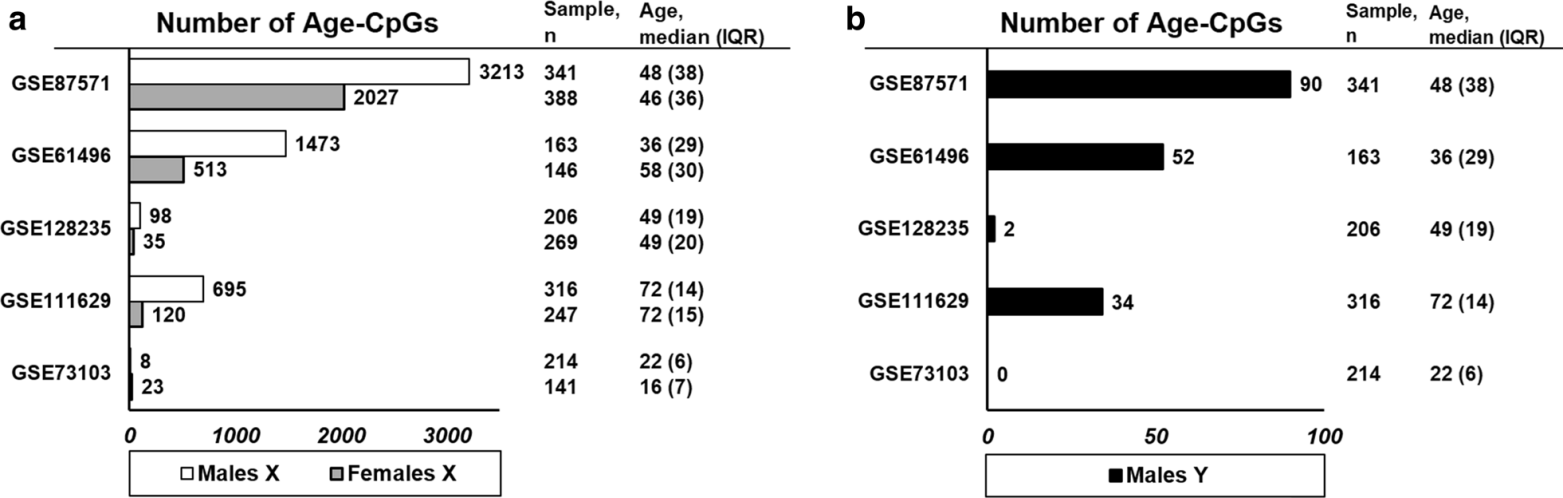
Characteristics of the study populations used and number of age-CpGs identified. Age distributions in the datasets are also visualized as histograms in Additional file 5: Fig. S1. Notably, the number of age-CpGs identified in each dataset was associated with the age range in the dataset. The data sets are shown in ranked order according the age interquartile range (IQR). Number of identified age-CpGs in each data set in males and females and in X (**A**) and Y chromosome (**B**) is visualized as bars. The number was higher in the X chromosome, as compared to the Y chromosome, reflecting the number of probes available for each chromosome on the Illumina 450 K array (11648 X chromosomal probes, 416 Y chromosomal probes). *n* = number of individuals in the data set

Of the five studied datasets, we identified age-associated CpG sites (hereafter referred as age-CpGs) in the Y chromosome in four datasets. The number of age-CpGs varied from 2 to 90 across datasets (Fig. 1). All Y chromosomal age-CpGs can be found from Additional file 1: Table S1A–D. There was considerable overlap between the datasets, as in all but one pairwise comparison, there was more overlap than can be expected by chance (Table 1). Of the identified CpG sites, 46 were age-associated in two or more datasets with corresponding direction of association (Additional file 1: Table S1E). As there are 416 Y chromosomal probes on the Illumina 450 K array, 11% of probes were identified as age-associated. Two CpGs were identified in four datasets (Table 2) and 14 CpGs in three datasets.

**Table 1:** Overlap of age-CpGs between data sets used in the study

	GSE61496	GSE73103	GSE87571	GSE111629
Male Y				
GSE73103	na			
GSE87571	7.17e-9 (34)	na		
GSE111629	4.22e-7 (18)	na	4.15e-9 (26)	
GSE128235	0.030 (2)	na	ns (2)	0.013 (2)
Male X				
GSE73103	ns (2)			
GSE87571	3.07e-269 (1099)	ns (5)		
GSE111629	1.04e-164 (406)	ns (1)	3.71e-146 (553)	
GSE128235	5.15e-25 (60)	ns (1)	1.32e-33 (90)	1.09e-24 (44)
Female X				
GSE73103	0.007 (5)			
GSE87571	3.13e-51 (259)	ns (5)		
GSE111629	3.24e-16 (34)	ns (1)	1.50e-30 (84)	
GSE128235	1.29e-06 (11)	0.003 (2)	1.82e-8 (23)	3.21e-10 (9)

In the table, the hypergeometric test *p* value for the overlap and the number of common age-CpGs in parentheses are presented. *na* not available due to no significant age-CpGs in GSE73103, *ns* not statistically significant, hypergeometric test *p* value > 0.05

**Table 2:** Age-CpGs identified in four or more datasets in one sex (*only cg25140188 identified in all five datasets studied). In boldface, age-CpGs identified in both females and males (in two or more datasets for the other sex)

	ID	Change with age	Gene (hg19)	Median β, males	Median β, females	CpG island
Y	cg27214488	Hyper	*NLGN4Y*	0.12		Shore
	cg20401549	Hypo	*PRKY*	0.07		Island
Male X	**cg12737514**	**Hyper**	***BCOR***	0.27	0.18	Island
	cg16140952	Hyper	*CHRDL1*	0.14		Shore
	cg21480420	Hyper	*FAM70A*	0.27		Shore
	cg06711837	Hyper	*FGF13*	0.15		Shore
	cg08783090	Hyper	*GABRE*	0.19		Island
	cg01222180	Hyper	*GPR50*	0.28		Island
	cg21672057	Hyper	*KIAA2022*	0.27		Island
	cg01289637	Hyper	*LRCH2*	0.09		Shore
	cg24823082	Hyper	*MID1IP1*	0.08		Shelf
	**cg26481961**	**Hyper**	***MXRA5***	0.19	0.17	Island
	cg00575851	Hyper	*NCRNA00087*	0.15		Shore
	cg12848223	Hyper	*NRK*	0.30		Shore
	cg14951132	Hyper	*NRK*	0.28		Island
	cg09935073	Hyper	*PAK3*	0.21		Open sea
	cg13775533	Hyper	*PCDH19*	0.37		Island
	cg05134041	Hyper	*POU3F4*	0.30		Shore
	cg09610569	Hyper	*SLITRK2*	0.11		Island
	cg10288278	Hyper	*SLITRK4*	0.30		Island
	**cg01538344**	**Hyper**	***TAF7L***	0.46	0.49	Island
	cg27326620	Hyper	*ZC4H2*	0.43		Open sea
	cg00525383	Hyper	*ZIC3*	0.23		Island
	cg03671371	Hyper		0.18		Island
	**cg03718079**	**Hyper**		0.18	0.35	Island
	**cg17156570**	**Hyper**		0.38	0.51	Shore
	**cg03660876**	**Hypo**	***ARHGAP6***	0.74	0.80	Open sea
	**cg18422972**	**Hypo**	***BCOR***	0.52	0.32	Island
	cg06758848	Hypo	*FLNA*	0.33		Island
	cg09761247	Hypo	*IDS*	0.19		Shore
	**cg00168417**	**Hypo**	***KIAA1210***	0.73	0.69	Shore
	cg03675615	Hypo	*PAGE4*	0.91		Open sea
	**cg25863147**	**Hypo**	***PDK3***	0.65	0.63	Shore
	**cg09641151**	**Hypo**	***PLXNA3***	0.67	0.44	Shore
	**cg04532200**	**Hypo**	***PLXNB3***	0.62	0.64	Shore
	cg19406003	Hypo	*PLXNB3*	0.42		Shelf
	cg18478786	Hypo	*SLC25A5*	0.11		Island
	cg21019788	Hypo	*SUV39H1*	0.28		Island
	**cg06461462**	**Hypo**	***TSC22D3***	0.66	0.75	Shore
	cg21900764	Hypo	*UPF3B*	0.09		Island
	cg19570406	Hypo	*ZFP92*	0.71		Shore
	cg25140188*	**Hypo**		0.44	0.46	Shelf
Female X	**cg01538344**	**Hyper**	***TAF7L***	0.46	0.49	Island
	**cg08704539**	**Hyper**	***TAF7L***	0.58	0.55	Island
	cg07811386	Hypo	*CA5B*		0.35	Open sea
	**cg00168417**	**Hypo**	***KIAA1210***	0.73	0.69	Shore
	cg05204193	Hypo	*MAP3K7IP3*		0.49	Shelf
	**cg04532200**	**Hypo**	***PLXNB3***	0.62	0.64	Shore
	cg02991082	Hypo	*TSC22D3*		0.10	Shore
	**cg06461462**	**Hypo**	***TSC22D3***	0.66	0.75	Shore
	cg17938879	Hypo			0.37	Open sea

Median methylation level across all datasets is given for both males and females

Majority, 76%, of the Y chromosomal age-CpGs identified in two or more datasets were hypermethylated. Their chromosomal distribution in relation to CpG islands was not different to what would be expected by chance. Regarding gene regions, we detected an enrichment of age-CpG sites in gene bodies and a depletion in intergenic regions (Additional file 1: Table S1F). Location of age-CpGs across the Y chromosome is presented in Additional file 5: Fig. S3. The 46 CpG sites were located in 18 genes (Additional file 1: Table S1E), but these genes were not enriched in any GO term categories, when Y chromosomal genes that were represented by one or more probes in the Illumina HumanMethylation450 BeadChip were used as background.

### X chromosome in males

We detected age-CpGs in the X chromosome in males in all five data sets studied, and the number of age-CpGs varied from 8 to 3213 across datasets (Fig. 1). All age-CpGs can be found from Additional file 2: Table S2A-E. There was considerable overlap between the age-associated CpGs in each dataset, as there was more overlap than can be expected by chance in the majority pairwise comparisons, with the exception of GSE73103, where we identified only 8 age-CpGs (Table 1). Of the identified CpGs, 1327 were identified as age-associated in two or more datasets with corresponding direction of association (Additional file 2: Table S2F). As there are 11 233 X chromosomal probes on the Illumina 450 K array, 12% of probes were identified as age-associated. Of these, one CpG (cg25140188) was identified in all five datasets and 39 CpGs were identified in four datasets (Table 2).

The identified age-CpGs were both hyper- (47%) and hypomethylated (53%) with age. The age-CpGs were enriched in CpG islands and depleted from open sea and island shelfs. Regarding gene regions, the age-CpGs were enriched in 1st Exon and 5’UTR but depleted from gene body, 3’UTR and from intragenic regions (Additional file 2: Table S2G). Location of age-CpGs across the X chromosome is presented in Additional file 5: Fig. S3. The 1327 age-CpGs were located in 491 genes (Additional file 2: Table 2F) and these genes were enriched in 47 GO terms, when X chromosomal genes that were represented by one or more probes in the Illumina HumanMethylation450 BeadChip were used a background (Additional file 2: Table 2H). For the most part, the identified GO terms were associated with cellular component organization and signal transduction.

### X chromosome in females

We detected age-CpGs in the X chromosome in females in all five datasets studied, and the number of age-CpGs varied from 23 to 2027 across datasets (Fig. 1). All age-CpGs can be found from Additional file 3: Table S3A-E. There was considerable overlap between the age-associated CpGs in each dataset, as there was more overlap than can be expected by chance in the majority pairwise comparisons, with the exception of GSE73103 (Table 1). Of the identified CpGs, 325 were identified in two or more datasets with corresponding direction of association (Additional file 3: Table S3F). As there are 11 233 X chromosomal probes on the Illumina 450 K array, 3% of probes were identified as age-associated. Of these, 9 CpGs were identified in four datasets (Table 2) and 29 in three datasets.

The identified age-CpGs were both hyper- (54%) and hypomethylated (46%) with age. As compared to males, the proportion of hypermethylated age-CpGs was higher and this difference was statistically significant (*p* < 0.05). The age-CpGs were enriched in CpG islands and depleted in open sea and CpG island shelfs. In relation to genes, the age-CpGs were enriched in TSS200 and depleted in gene body (Additional file 3: Table S3G). Location of age-CpGs across the X chromosome is presented in Additional file 5: Fig. S3. The 325 age-CpGs were located in 199 genes (Additional file 3: Table S3F), and these were enriched in two GO component terms, when X chromosomal genes that were represented by one or more probes in the Illumina HumanMethylation450 BeadChip were used as background (GO0044464, *cell part*, FDR q value = 0.003; GO0016020, *membrane*, FDR q value 0.003).

### Overlap between males and females in the X chromosome

Of the 1327 and 325 X chromosomal age-CpGs identified in males and females, respectively, 122 were identified in both sexes with corresponding direction of association and additional 5 were identified in both sexes but with opposite direction of association (Additional file 4: Table S4A). Overlap of CpGs with comparable direction of association was more than would be expected by chance (hypergeometric test, *p* value = 1.1e-35). Four CpGs (cg00168417, cg01538344, cg04532200, and cg06461462) were identified in four datasets in both females and males (Table 2).

Of the age-CpGs identified in both sexes, 57% were hypermethylated with age. The age-CpGs common for males and females were depleted from open sea as well as from gene bodies (Additional file 4: Table S4B). The 122 age-CpGs were located in 84 genes (Additional file 4: Table S4A), that were enriched in one GO process term (GO0071840: *cellular component organization or biogenesis*, FDR q value = 0.049), when X chromosomal genes that were represented by one or more probes in the Illumina HumanMethylation450 BeadChip were used as background.

## Discussion

To gain insight on age-associated changes in DNA methylation in sex chromosomes, we utilized whole blood methylation data available in data repositories and report results from five individual datasets, comprising of ~ 2400 individuals aged 14–92 years.

The age-CpGs identified in the five datasets showed significant overlap across the datasets. In X in both sexes, and in Y in males, the number of age-CpGs was dependent on the age range of the sample; the wider the range, the more age-CpGs were detected (Fig. 1). In the X chromosome, we identified more significant age-CpGs in males as compared to females. Despite the difference in number of age-CpGs and the uneven chromosome dosage, the X chromosomal age-CpGs showed significant overlap between females and males and displayed similar characteristics in terms of genomic locations and direction of change. These age-CpGs were enriched in CpG islands and towards the 5’ end of genes but depleted from gene bodies. For the X chromosome, we did not observe a strong trend towards hyper- or hypomethylation. The proportion of hypermethylated age-CpGs was higher in females as compared to males, but this difference can be affected by the different number of age-CpGs identified in females and males (325 and 1327, respectively). Age-CpGs in the Y chromosome, however, were predominantly hypermethylated with age and contrary to the X chromosomal age-CpGs, were enriched in gene bodies.

Ageing is associated with a global hypomethylation of the genome [13], and in the autosomes, majority of studies on age-associated changes in DNA methylation have identified more hypomethylated age-associated sites as compared to hypermethylated [10, 14–21]. Notably, some studies do report more hypermethylated age-associated sites [22–25]. For the X chromosome, Li et al. (2020) [26] reported a trend towards hypermethylation for female-specific age-associated CpGs and inconsistent results for male-specific CpGs. In our own results, we also identified more hypermethylated age-CpGs in females, but the trend was not as strong as reported by Li et al. [26]. For age-associated X chromosomal probes identified in both sexes, they reported a strong trend towards hypermethylation (78–94% of identified CpGs hypermethylated), similar to the more modest trend of hypermethylation we observed for X chromosomal age-CpGs observed in both sexes (57%). For the Y chromosome, Lund et al. (2020) [27] reported a strong trend towards hypermethylation with age (> 82% hypermethylated), and we observe a similar trend in our analyses (76% hypermethylated). This suggests that the age-associated changes in DNA methylation especially in the Y chromosome are distinct from those in the autosomes. However, as the number of Y chromosomal probes on the Illumina 450 K array is considerably lower as compared to X chromosomal probes (416 versus 11 233 probes, respectively), these results should be interpreted with caution.

GO term enrichment analysis of the sex-chromosomal age-CpGs did not associate these CpGs with functions that are known to be strongly associated with the molecular mechanisms of ageing [28]. The individual age-CpGs identified were located in genes with various functionalities. Of the individual age-CpGs, cg25140188 was identified as age-associated in males in all five datasets studied, in females in two of the datasets studied, and previously by McCartney et al. (2020) [29]. However, this CpG is not annotated with a gene. Four X chromosomal CpG sites were identified as age-associated in four datasets in both females and males, and each of these were located within a protein coding gene. Two of these, *KIAA1210* (cg13498184) and *TAF7L* (TATA-box-binding protein associated factor 7 like) (cg01538344), are mainly expressed in testes and have been implicated to have a role in spermatogenesis [30, 31]. *TAF7L* is also classified as a cancer-testis antigen (CTAg), belonging to a group of genes typically expressed only in the germline but also in malignant tumours [32]. Two additional CpG sites located in *TAF7L* were also identified as age-CpGs in at least two datasets in both females and males included in this study (Additional file 4: Table S4A). *PLXNB3* (plexin B3) (cg04532200) is highly expressed in the brain and plays a role in axon guidance [33]. *TSC22D3* (TSC22 domain family member 3) (cg06461462) regulates T-cell activation and has immunosuppressive and anti-inflammatory effects [34]. Dysregulation of the immune system, the so-called immunosenescence, is an important feature of the ageing phenotype [35].

The two Y chromosomal CpGs that were identified in four of the five studied datasets are located in *PRKY* (cg20401549) and *NLGN4Y* (cg27214488). One additional CpG site located in *PRKY* and seven additional CpG sites located in *NLGN4Y* were also identified as age-CpGs in at least two datasets included in this study (Additional file 1: Table S1E). *PRKY* is a protein kinase pseudogene, and there is a similar gene in the pseudoautosomal region of the X chromosome. Interestingly, three a-CpGs identified in both females and males were located in *NLGN4X* (neuroligin 4 X-linked) (Additional file 4: Table S4A). *NLGN4X* and *NLGN4Y* share 97% sequence identity, but a difference of only one amino acid leads to deficit in *NLGN4Y* function, as compared to *NLGN4X* [36]. Neuroligins are associated with neuronal development and synaptic transmission, and they have been linked with autism spectrum disorders and intellectual disability [37]. In addition, *NLGN4Y* has been suggested to be associated with male homosexuality [38] and prostate cancer [39]. A CpG (cg01707559) identified previously by Lund et al. [27] and replicated in the present study is located in *TBL1Y* (transducin beta like 1 Y-linked), a gene that is highly expressed in the prostate and shares great similarity with *TBL1X* in the X chromosome. We also identified two additional a-CpGs located in *TBL1Y* in the present study (Additional file 1: Table S1E).

In most previous studies on age-associated DNA methylation changes, sex chromosomes have been excluded from the analyses [9, 10, 14–18, 23, 24, 40–44]. Other studies, while not explicitly stating whether or not sex chromosomes have been included in the analyses, report no significant findings in X or Y chromosomes related to age [11, 12, 19, 21, 22, 25, 45]. However, while majority of studies have not explored this, it should be noted that also autosomes can display sexually dimorphic age-associated changes in DNA methylation [46].

Only a handful of studies, that have not specifically focused on sex chromosomes, including only women, report significant age-associated findings in the X chromosome [20, 47, 48]. Each of these studies report five or less age-associated CpGs in the X chromosome, none of which are replicated between these three studies. Of these, one CpG site (cg27250462) reported by Teschendorff et al. [20] is replicated in a study focusing on age-associated methylation changes in the X chromosome (Li et al. 2020) [26], and one CpG site (cg13277716) reported by Xu & Taylor (2014) [48] is replicated in both females and males in three of the datasets analyzed in this study (GSE111629, GSE61496, GSE87571).

Some studies on age-associated DNA methylation changes have included X chromosomal probes in the analyses, but processed and analyzed female and male samples together [29, 49, 50]. Of these, Jansen et al. (2019) [50] report no significant age associations in the X chromosome, whereas Kim et al. (2014) [49] report an ageing-signature that is highly enriched to the X chromosome. As the statistical analyses were not sex-stratified, the findings might be biased due to differential methylation profile in female and male X chromosomes. In the study by McCartney et al. (2020) [29], sex and sex*age interaction were included in the analysis, and among their high-confidence age-associated CpGs, they report 5 sex-independent and 6 sex-dependent X chromosomal sites. Of the sex-independent CpGs, one (cg25140188) was identified as age-CpG in both females and males in this study, and in males in all of the five datasets studied. Of the sex-dependent CpGs reported by McCartney et al. (2020) [29], one CpG (cg20202246) was identified as age-CpG in both females and males and another CpG (cg08814148) only in males.

Only a few studies specifically focusing on age-associated DNA methylation changes in X or Y chromosomes have been published. Cotton et al. (2015) [51] reported no age-associated CpGs in the X chromosome using a small dataset (n = 111). More recently, Li et al. (2020) [26] have studied ageing-associated changes in DNA methylation in the X chromosome in two discovery datasets, and a third replication dataset. They identified 27 CpGs in females and 19 CpGs in males as age-associated in all three datasets in their study. In our study, in females, none of the 27 CpGs reported by Li et al. [26] were identified as age-CpGs, whereas in males, 4 CpGs out of the 19 reported by Li et al. were identified as age-CpGs with similar direction of change with age (Table 3).

**Table 3:** Age-CpGs in male X chromosome identified in Li et al. (2020) [26] and in the present study, all hypermethylated with age

ID	Data sets in this study	Gene
cg05070565	GSE61496, GSE87571	*WAS*
cg15222604	GSE111629, GSE61496, GSE87571	*HCFC1*
cg18086582	GSE61496, GSE87571	*WDR13*
cg21120047	GSE61496, GSE87571	*XPNPEP2*

One study only has previously focused specifically on ageing-associated DNA methylation changes in the Y chromosome (Lund et al. 2019) [27]. In total, they utilized four datasets, and report 7 CpGs that were consistently hypermethylated with age across all data sets. Of these, one (cg01707559), located in *TBL1Y*, is replicated in our study in three datasets (GSE111629, GSE61496, GSE87571).

In genome-wide DNA methylation studies on autosomes, at the level of individual CpGs, the results have not shown great replication from study to study. Only a small number of CpG sites, for example those located in *ELOVL2*, *FHL2,* and *EDARADD*, have been replicated in majority of studies [10, 20, 25, 40, 42, 45, 48, 52]. This pattern of small accordance between studies is similar for the sex chromosomes, as a very limited number of CpGs are identified as age associated in more than one study. Presumably, both sample and dataset characteristics as well as data processing and analysis methods contribute to this discrepancy.

Specifically, age distribution within and between datasets likely influences the replication of age-CpGs. In our analysis, the number of age-CpGs was dependent on the age distribution of the dataset, with higher the IQR for age, the higher the power was to detect significant age-CpGs (Fig. 1). Previously, variation in DNA methylation has been shown to increase with age for both autosomes [42, 53], and the Y chromosome [27]. Modest variation in DNA methylation at younger adult ages, and low IQR for age, may explain why we did not identify many changes in dataset GSE73103. Furthermore, the study by Li et al. (2020) [26] consisted of older individuals aged > 55 years, which may in part explain the low accordance with our results of X chromosomal age-CpGs.

To decipher to what extent data processing methods could explain the limited overlap between our results and those reported by Li et al. (2020) [26], we repeated our analysis in a data set with the highest number of age-CpGs (GSE87571) as Li et al. [26] had done. While both age-association analyses were performed for X chromosomal probes only, Li et al. [26] excluded autosomal probes before normalization, whereas in our study, the autosomal probes were excluded from the analysis only after normalization. However, this did not change our results for dataset GSE87571, and the overlap with Li et al. remained limited, further suggesting that dataset and sample characteristics might explain the observed discrepancies. Further details of this additional analysis can be found from Additional file 5.

The sex chromosomes can also undergo more large-scale changes with age, which could affect the observed DNA methylation pattern. Both loss of Y (LOY) and loss of X (LOX) have been associated with ageing [54, 55]. Especially, LOX could affect the observed DNA methylation pattern, as the fully methylated, inactive X is more often affected by LOX [55]. In the present study, we cannot exclude that some of the age-associated changes observed are actually due to LOX. Skewing in the proportion of inactivated X has also been suggested to be associated with ageing [56–58]. Several papers catalogue human X chromosomal genes escaping from XCI [51, 59, 60]. The X chromosomal age-CpGs in females in this study mapped to 199 protein coding genes. Of these, 48 were classified as escaping XCI in these three studies [51, 59, 60], so in total, 25% of genes harboring age-CpGs in this study have been implicated to escape XCI. Total percentage of X chromosomal genes escaping XCI is considered to be approximately one third of all X chromosomal genes [51, 60], thus suggesting that genes escaping XCI are not overrepresented among those harboring age-associated DNA methylation changes. However, Li et al. (2020) [26] reported a much lower proportion of XCI escape, approximately 6% of all genes in the X chromosome (648 CpGs located in 133 genes). Of the 232 age-CpGs in females identified in this study, 55 (17%) were described as escapees by Li et al. (2020) [26], suggesting an overrepresentation of escapees among the genes harboring age-CpGs. Taken together, age-associated DNA methylation changes in the X chromosome occur in both XCI escaping genes and those that remain inactivated.

A limitation of our study is that as we used data from a data repository, we had only limited phenotypic information that could be adjusted for in the analysis. Adjusting for confounding factors such as disease status or lifestyle factors could increase the sensitivity of the analysis to identify age-CpGs. We did adjust for cell-type proportions, as these can be estimated from the methylation data itself, and they have been shown to explain majority of variation in DNA methylation [61].

## Conclusion

Taken together, we report here ageing-associated changes in DNA methylation in both X and Y chromosomes. Our results show the similarities in age-associated DNA methylation changes in females and males and suggest that they share features with DNA methylation changes observed in the autosomes. Significant overlap in X chromosomal age-CpGs identified in males and females and their shared features suggest that despite the uneven chromosomal dosage, differences in ageing-associated DNA methylation changes in the X chromosome are unlikely to be a major contributor of sex dimorphism in ageing. Age-associated DNA methylation changes in the Y chromosome appear to differ from those observed in the autosomes, but due to technical reasons, the results should be interpreted with caution.

Very little has been published regarding the age-associated DNA methylation changes in the sex chromosomes and the published results show very limited overlap. Future study is needed to pinpoint analysis methods that yield biologically the most significant results. In addition, functionality of the observed DNA methylation changes in the sex chromosomes and more broadly throughout the genome requires more study. Analysis strategies, including for example pathway and enrichment analysis, that allow the combination of results from autosomes and sex chromosomes could possibly produce the most accurate picture of these changes. In addition, and especially for the Y chromosome, DNA methylation analysis with, for example, a bisulphite-sequencing method with better coverage could help to differentiate true biological phenomenon from technical bias.

## Materials and methods

### Datasets

Methylation data were downloaded from GEO NCBI database [62] using the following inclusion criteria: number of individuals in the dataset was > 200, information on age and sex was available, sample type was whole blood, DNA methylation was determined using Illumina HumanMethylation450 BeadChip, and raw data were available as idat-files. Data sets included were GSE61496 [63], GSE73103 [64], GSE87571 [15], GSE111629 [65], and GSE128235 [66]. Datasets are further described in Fig. 1 and in Additional file 5: Fig. S1.

GSE61496 consists of monozygotic twin pairs from Danish Twin Registry, who are disconcordant for birth weight. GSE73103 consists of healthy individuals recruited in Uppsala, Sweden. GSE87571 is a sample of The Northern Sweden Population Health Study in Sweden. GSE111629 is a sample of a population-based case–control study of Parkinson’s disease in California, USA. GSE128235 is comprised of depressed and control subjects who were recruited at the Max Planck Institute of Psychiatry, Germany. Detailed information can be found under accession numbers at GEO NCBI database [62].

### Processing of methylation data

For each dataset, male and female samples were preprocessed separately using R software and minfi therein. Visual inspection of the data (Additional file 5: Fig. S2) shows the different distribution of methylation values for female and male X chromosome, consistent with XCI in females, demonstrating the rationale of analyzing male and female samples separately. Correct assignment of sex in the phenotypic data for each dataset was verified by the median methylation value of the X chromosome.

From each dataset, multimapping and cross-reactive probes, probes with detection p value median  > 0.05 and probes with minor allele frequency of 1% or higher [67] were excluded. Annotation was hg19. The datasets were background corrected and quantile normalized in minfi, and dye bias was corrected with BMIQ in wateRmelon. Methylation profiles after preprocessing are shown in Additional file 5: Fig. S2.

Individual samples were excluded based on low methylated and unmethylated signals with a cut-off value for the average level at 10.5. With this criterion, we excluded 60 samples from GSE128235 (23 male samples, 37 female samples) and one male sample from both GSE61496 and GSE111629. Individual methylation level values were excluded when detection p value was more than 0.05. After preprocessing, the number of probes remaining for statistical testing across datasets was 9505–9513 for female X, 9717–9807 for male X, and 296–298 for male Y.

Methylation sites associated with age in X and Y chromosomes were identified within each data set using linear multivariate modeling. Methylation level, ß-value, was set as the dependent variable and the model was adjusted for blood cell composition. The blood cell composition was estimated using function EstimateCellCounts. Both twins in GSE61496 were included and considered as singletons in the analysis. Threshold for statistical significance within each data set was Benjamini–Hochberg-adjusted *p* value < 0.05.

### Characterizing, visualizing, and comparing datasets

Overlap between datasets in this study and other studies were visualized using webtool Venn [68]. Chromosomal ideograms were produced using PhenoGram software [69]. Statistical significance of overlap was analyzed with hypergeometric test. Enrichment of GO terms was analyzed with GOrilla [70, 71]. Only X and Y chromosomal genes that were represent by one or more probes in the Illumina HumanMethylation450 BeadChip were used as background in the analyses.

## Supplementary Information

**Additional file 1:** Y chromosomal age-CpGs. Contains lists of Y chromosomal age-CpGs identified in each individual dataset (**A–D**), list of age-CpGs identified in multiple datasets (**E**) and location of identified Y chromosomal age-CpGs in relation to CpG islands and gene regions (**F**).

**Additional file 2:** X chromosomal age-CpGs identified in males. Contains lists of X chromosomal age-CpGs identified in males in each individual dataset (**A–E**), list of age-CpGs identified in multiple datasets (**F**), location of identified X chromosomal age-CpGs in relation to CpG islands and gene regions (**G**) and enriched GO terms for X chromosomal age-CpGs in males (**H**).

**Additional file 3:** X chromosomal age-CpGs identified in females. Contains lists of X chromosomal age-CpGs identified in females in each individual dataset (**A–E**), list of age-CpGs identified in multiple datasets (**F**) and location of identified X chromosomal age-CpGs in relation to CpG islands and gene regions (**G**).

**Additional file 4:** X chromosomal age-CpGs identified in both females and males. Contains list of X chromosomal age-CpGs identified in both females and males (**A**) and location of those age-CpGs in relation to CpG islands and gene regions (**B**).

**Additional file 5:** Figures and Supplementary analysis.

## Data Availability

The datasets supporting the conclusions of this article are available in the Gene Expression Omnibus (GEO, www.ncbi.nlm.nih.gov/geo/) repository, under accession numbers GSE61496, GSE73103, GSE87571, GSE111629, and GSE128235.
