# Supplementary material for: Ageing-associated changes in DNA methylation in X and Y chromosomes
Source: Epigenetics Chromatin. 2021 Jul 2;14:33. doi: 10.1186/s13072-021-00407-6 (PMC8254238; doi:10.1186/s13072-021-00407-6)
Supplement: Supplementary file 5 — Additional file 5: Figures and Supplementary analysis. [file 13072_2021_407_MOESM5_ESM.docx]

**Additional file 5**


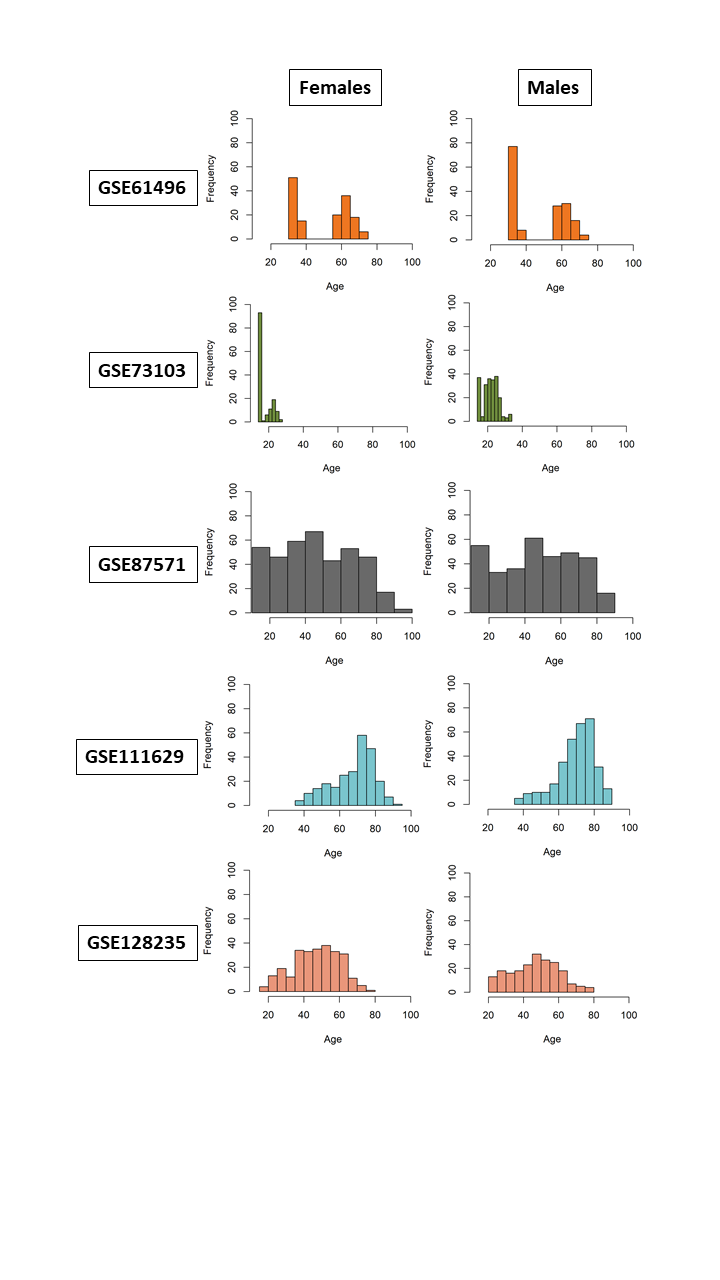


**Figure S1.** Age distributions in the data sets used in the analysis.

**
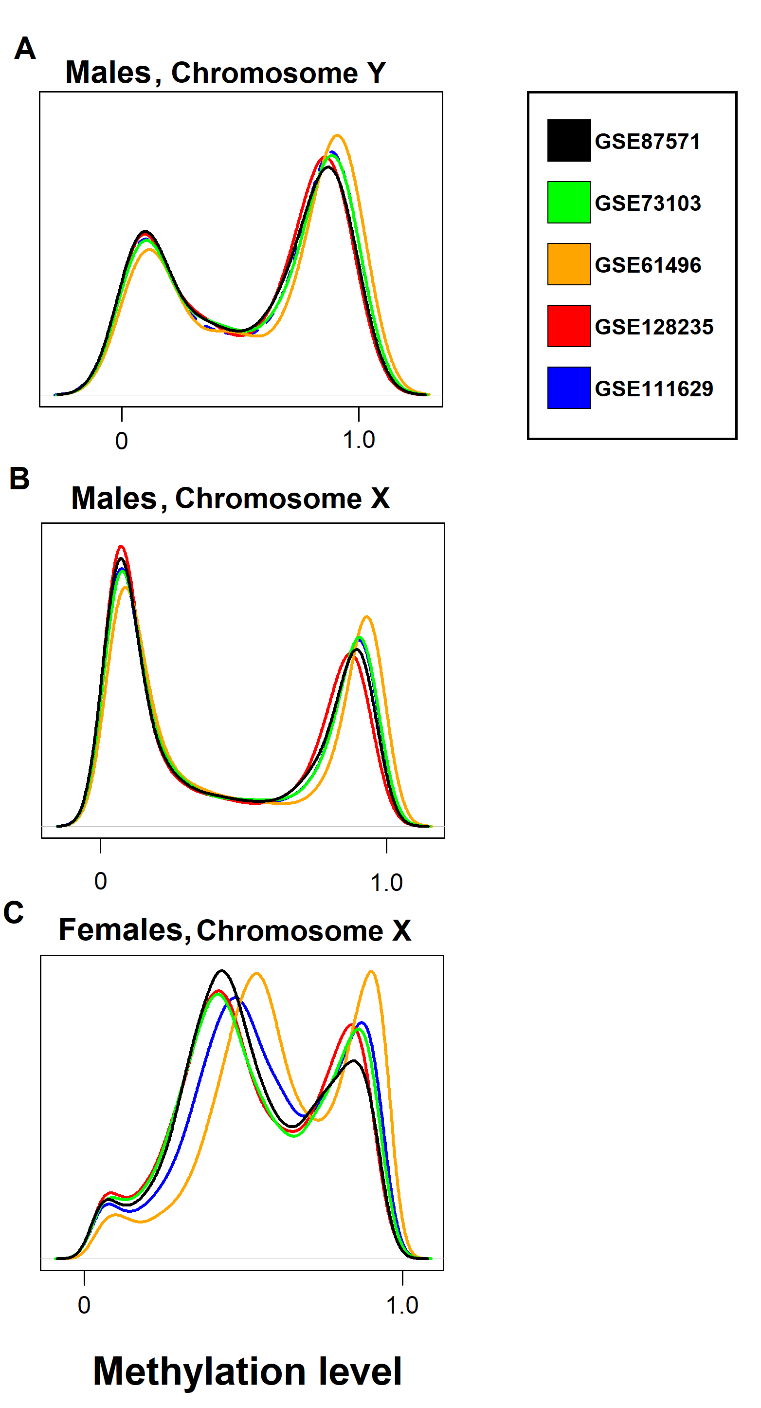
**

**Figure S2.** Methylation level medians in each methylation site in chromosome Y in males (A), in chromosome X in males (B), and in chromosome X in females (C) visualised as density plots.

**
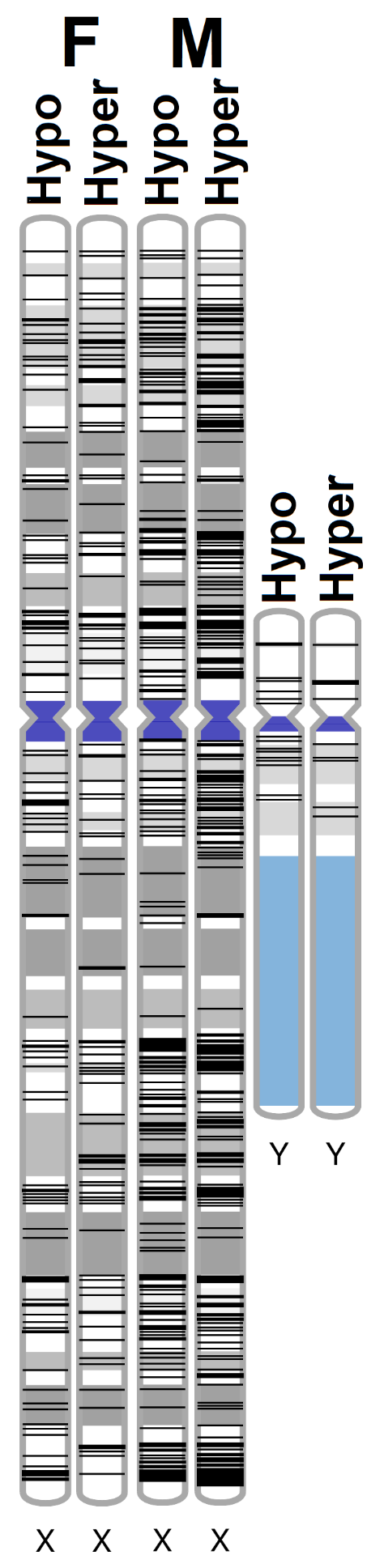
**

**Figure S3. Physical locations of age-CpGs in the X chromosome in females (F) and in the X and Y chromosome in males (M).** Genomic locations (black lines) are shown separately for age-CpGs identified in at least two data sets where methylation increases (hyper), and decreases (hypo) with advancing age. Cytobands are indicated in blue and grey coloring.

**Analysis S1**

*Comparison of two DNA methylation data preprocessing approaches in chromosome X in females*

Two preprocessing approaches were tested in DNA methylation data of females in GSE87571 (n=388, aged 14-94 years). In normalization version 1, all DNA methylation sites in the genome were preprocessed and normalized as one data set before statistical testing, and in normalization version 2, methylation sites in X chromosome were preprocessed after excluding other sites locating in autosomes. Otherwise the preprocessing pipeline was the same as described in the methods in the main text.

As shown in Supplementary Figure 3, within the 2027 ageing-associated methylation sites in X chromosome in females in GSE87571 (original analysis, normalization v1), the directions of methylation change with age, represented by the regression coefficients were the same in the analysis where the preprocessing was performed using normalization version 2.

Of the 2027 ageing-associated methylation sites identified from data processed using normalization v1, 1692 were associated with age (BH-adjusted p-value<0.05) also when using normalization version 2. When comparing the results from normalization version 1 and 2 in GSE87571 to those by Li et al., the overlap was similar for both normalization versions. In addition, e.g. the four CpGs (cg00168417, cg01538344, cg04532200, cg06461462) in chromosome X that were associated with age in both sexes and in four data sets processed using normalization v1, were all associated with age also in data processed using normalization v 2. This indicates that the majority of differences in the analyses does not arise from decision to include or exclude autosomes in preprocessing steps.


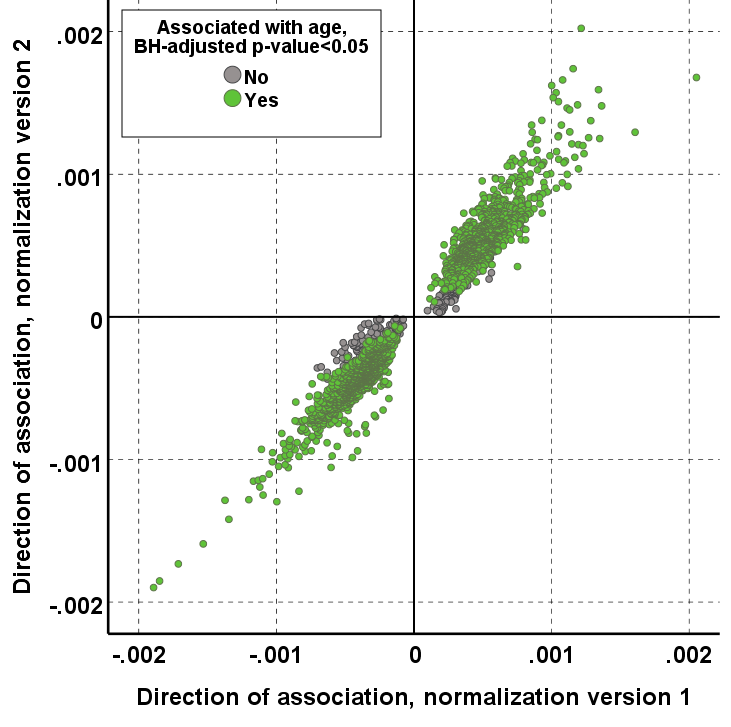


**Figure S3. Comparison of two DNA methylation data preprocessing approaches in chromosome X in females (GSE87571).** Directions of associations with age of the methylation sites from the preprocessing pipeline with 1) normalization version 1 (all DNA methylation sites in the genome were preprocessed and normalized as one data set before statistical testing) and 2) normalization version 2 (only DNA methylation sites in chromosome X were preprocessed and normalized before statistical testing) were compared. This comparison is visualised as scatterplot. (Direction of association = regression coefficient)
